# Colorectal Cancer Burden and Access to Federally Qualified Health Centers in California

**DOI:** 10.5888/pcd12.150162

**Published:** 2015-10-08

**Authors:** Brendan Darsie, Jennifer Rico, Madhurima Gadgil, Joshua Tootoo

**Affiliations:** Author Affiliations: Jennifer Rico, Madhurima Gadgil, Chronic Disease and Injury Control Division, California Department of Public Health, Sacramento, California; Joshua Tootoo, Children’s Environmental Health Initiative, University of Michigan, Ann Arbor, Michigan.

**Figure:**
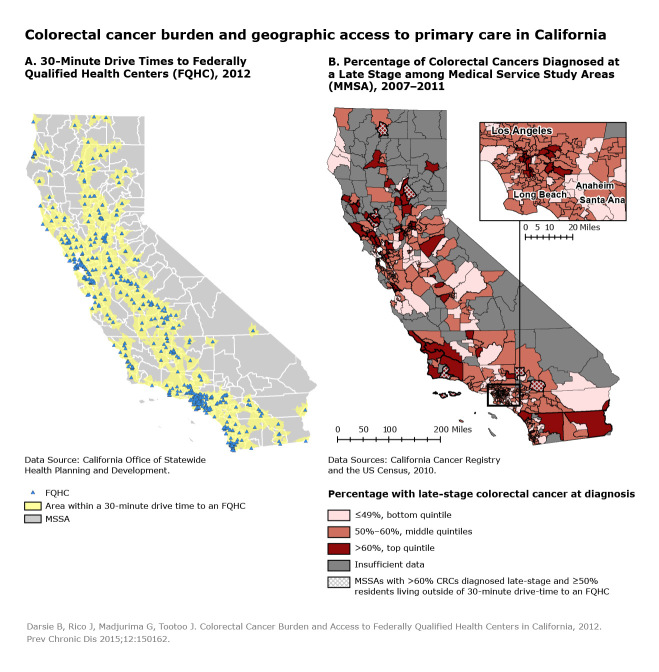
Colorectal cancer burden and access to primary health care, California. A vast majority of Californians (96%) live within a 30-minute drive of a federally qualified health center (FQHC). This is true for areas in both the top and bottom quintiles (96% and 93% respectively) based on the percentage of colorectal cancers (CRC) diagnosed at a late stage. There was no meaningful connection found between geographic access to affordable CRC screening services and late-stage diagnosis percentages. This finding suggests that other barriers besides physical distance to affordable CRC screening need to be examined in order to reduce the geographic disparities in late-stage CRC diagnoses.

## Background

Colorectal cancer (CRC) is the third most common cancer in California among both men and women and the third most common cause of cancer-related death (1). CRC mortality in California has declined over the past 25 years, due in part to increased screening rates (2). Early detection of CRC greatly increases survival, but more than 50% of people with CRC cases are diagnosed at a late stage (3). Californians diagnosed with CRC that is localized to the colon or rectum have a 95% 5-year survival rate (3). However, when CRC has spread to the lymph nodes, 5-year survival drops to 66%, and when the cancer has spread to distant organs, 5-year survival is only 12%. Thus, screening is essential to the early detection and successful treatment of CRC.

Socioeconomic status is a strong predictor of colorectal cancer screening (4). Studies have shown that people with low income and limited or no health insurance face many barriers to accessing health care services and screening for CRC (4). Increasing screening rates among low-income and underinsured people may significantly decrease the burden of CRC. Federally qualified health centers (FQHCs) are an important source of primary care for low-income and underinsured Americans. FQHCs receive approximately 40% of their funding from federal grants that mandate the provision of health care to an underserved population, the implementation of a sliding fee scale based on income, and the provision of comprehensive health services, including CRC screening (5). This GIS Snapshot examines geographic variation in the percentage of late-stage CRC diagnoses in California and the percentage of the population within a 30-minute drive time to an FQHC. 

## Methods

We selected for our analysis the 682 FQHCs and FQHC look-alikes (centers that serve the same population as FQHCs but do not receive the same grant funding) that were active in California in 2012. We measured geographic access (availability and accessibility of services) to FQHCs by using a spatial, street-level network analysis to calculate 30-minute drive-time buffers (6). Block-group–level population data and population-weighted centroids from the 2010 US Census were used to calculate the percentage of the California population living within these 30-minute drive-time areas (7). This percentage was our measurement of access to FQHCs. We used the percentage of late-stage diagnoses of CRC (regional and distant stages) by California’s Medical Service Study Areas (MSSAs) during 2007–2011, obtained from the California Cancer Registry, to describe the burden of CRC in California. MSSAs are the defined geographic analysis units for the California Office of Statewide Health Planning and Development. Beginning with the 2010 Census, there were 542 MSSAs in California. MSSAs are aggregations of census block groups and tracts and make up “rational service areas” for primary health care. We identified the quintiles of MSSAs with the highest and lowest percentage of CRCs diagnosed at a late stage and compared the residents’ geographic access to FQHCs. The top quintile consisted of MSSAs with more than 60% of CRCs diagnosed at a late stage; the bottom quintile consisted of MSSAs with 49% or fewer CRCs diagnosed at a late stage. We used a *z*-test to determine whether the top and bottom quintiles were significantly different in the proportion of their populations within a 30-minute drive of an FQHC. We also compared the proportion of households without access to a private vehicle in the top quintile versus the bottom quintile of late-stage CRC diagnosis by using data from the 2008–2012 American Community Survey (www.census.gov/programs-surveys/acs/). We considered *P* ≤ .05 significant. We used Esri’s ArcGIS version 10.2 for desktop (Esri) to create maps by applying the Network Analyst Extension. We used Esri’s Smart Data Compression network data set to perform the drive-time analysis. All other analyses were completed in SAS version 9.4 (SAS Institute, Inc).

## Findings

Most (96%) of California’s population was within a 30-minute drive of an FQHC (Figure, Map A). Ninety-six percent of the population of MSSAs in the top quintile of late-stage CRC diagnosis and 93% of the population in the bottom quintile of MSSAs were also within a 30-minute drive of an FQHC (Table). The difference between the percentages was statistically significant (*P* <.001), but this difference may not be practically important because the difference between them was only 3 percentage points. Compared with the bottom quintile, the top quintile had a greater percentage of people living below the poverty level, a higher percentage of people without access to a private vehicle, and a higher percentage of people living in rural MSSAs (Table). These differences may be contributing factors to the observed disparities in the percentage of people diagnosed with late-stage CRC in those areas.

**Table:** Characteristics of Californians, by Quintile of Diagnosis of Late-Stage Colorectal Cancer Among Medical Service Study Areas (MSSAs), 2010^a^

Characteristic	California	Residents in Bottom Quintile MSSAs (≤49%)	Residents in Top Quintile MSSAs (>60%)	*P* Value^b^
**Population, n**	37,253,956	6,389,178	4,186,101	NA
**Age >65 y**	11.4	10.6	11.5	<.001
**Race/ethnicity**				
Hispanic	37.6	33.8	37.8	<.001
Non-Hispanic Asian/Pacific Islander	13.2	13.1	11.6	<.001
Non-Hispanic black	5.8	4.2	5.2	<.001
Non-Hispanic white	40.1	45.9	42.6	<.001
Other	3.3	3.0	2.8	<.001
**Rural^c^**	13.1	18.3	23.8	<.001
**Income below federal poverty level^d^**	15.3	11.4	16.4	<.001
**No access by private vehicle^d^**	7.7	5.2	9.9	<.001
**Lives within 30-min drive-time of an FQHC**	95.9	93.2	96.3	<.001

Abbreviations: FQHC, federally qualified health center; NA, not applicable.

a Values are percentages unless otherwise indicated.

b *P* values were calculated by using *z* test for differences between top and bottom quintiles.

c Percentage of population living in rural MSSAs, which are defined as having a population density of fewer than 250 people per square mile and have no census-defined place within that area with a population exceeding 50,000 people (9).

d Data are from the 2008–2012 American Community Survey (www.census.gov/programs-surveys/acs/).

## Action

These maps illustrate the impressive geographic coverage that FQHCs have in California; more than 90% of Californians lived within a 30-minute drive of an FQHC. This is true for areas with the highest and lowest percentages of late-stage CRC. However, in 2012, the CRC screening rate of California FQHC patients eligible for CRC screening was only 34.9% (8). This finding suggests that other barriers besides physical distance to affordable CRC screening may exist. For example, a significantly greater proportion of households in the top quintile of late stage CRC diagnosis did not have access to a private vehicle (10%) compared with those in the bottom quintile of late stage CRC diagnosis (5%; *P* < .001) (Table). In addition, people in MSSAs close to an FQHC may not be aware of the availability of affordable CRC screening. Areas outside of the 30-minute drive time to an FQHC, especially areas with a high percentages of late stage CDC diagnoses, may particularly benefit from more CRC screening resources. Further research about the barriers to CRC screening is needed to reduce colorectal cancer mortality rates and increase the health and well-being of Californians.
